# Laparoscopic Resection in the Management of Non-adrenal and Non-renal Retroperitoneal Tumors: A Systematic Review

**DOI:** 10.7759/cureus.92403

**Published:** 2025-09-15

**Authors:** Alexis Quetzalcoatl Vega Morales, Patricio Xavier Duran S, Elisa Cohen Balas, Andrea Blanco Silva, Melanie Mishel Ponce Saldaña, Milton Fernando Crespo Macias, Yilber Andrés Motta Rojas, Raúl Albarrán Flores, Juan Sebastian Hernández Cedeño

**Affiliations:** 1 Specialization Program in General Surgery, Universidad de Guadalajara, Guadalajara, MEX; 2 General Surgery, Hospital General Regional No. 180 del Instituto Mexicano del Seguro Social (IMSS), Guadalajara, MEX; 3 Internal Medicine, Universidad de Cuenca, Cuenca, ECU; 4 Medicine, Universidad Anahuac México North Campus, Huixquilucan de Degollado, MEX; 5 Anesthesiology and Critical Care, Instituto Mexicano del Seguro Social (IMSS), Mexico City, MEX; 6 Medicine, Universidad de San Martín de Porres, Lima, PER; 7 General Medicine, Ministry of Health of Chile, Santiago, CHL; 8 General Medicine, Universidad Nacional de Colombia, Bogotá, COL; 9 General Medicine, Universidad de la Salud del Estado de México, San Cayetano Morelos, MEX; 10 General Medicine, Universidad Católica Santiago de Guayaquil, Guayaquil, ECU

**Keywords:** laparoscopic resection, paraganglioma, perioperative outcomes, retroperitoneal sarcoma surgery, schwannoma

## Abstract

Retroperitoneal tumors (RPTs) are rare, often presenting late due to their deep anatomical location. Complete surgical excision remains the mainstay of treatment. While open surgery is traditional, advances in minimally invasive techniques have expanded laparoscopic resection to selected non-adrenal, non-renal RPTs. Following the Preferred Reporting Items for Systematic Reviews and Meta-Analyses (PRISMA) 2020 guidelines, we systematically searched PubMed, ScienceDirect, Google Scholar, and Cochrane Library databases up to August 1, 2025. Eligible studies included adults undergoing laparoscopic resection for large-volume, non-adrenal, non-renal RPTs, reporting perioperative outcomes. Data extraction and ROBINS-I (Risk Of Bias In Non-randomised Studies - of Interventions) quality assessment were performed. Due to heterogeneity, results were synthesized narratively without meta-analysis. Across all included studies, laparoscopic RPT resection demonstrated favorable perioperative outcomes, with a few studies directly comparing it to open surgery and reporting superior perioperative results. Operative time varied, with some reports showing longer durations laparoscopically, though learning curves reduced times substantially. Blood loss was consistently lower in the laparoscopic group, often eliminating the need for transfusions. Complication rates were low, and conversion to open surgery was infrequent. Hospital stays were shorter with earlier ambulation and diet resumption in laparoscopic cases. Feasibility was highest in early-stage and benign tumors, with high completion rates. Oncologic outcomes were comparable, with low recurrence rates in well-selected patients. These findings support the safety, efficacy, and minimally invasive advantages of laparoscopic management for RPTs in appropriately selected cases. Laparoscopic RPT resection offers reduced morbidity, faster recovery, and comparable oncologic outcomes compared to open surgery, making it a safe and effective option in selected patients and experienced surgical settings.

## Introduction and background

An uncommon and varied category of neoplasms that develop in the retroperitoneal area, retroperitoneal tumors (RPTs) make up about 0.1-0.2% of all malignancies [1,2]. Because of the retroperitoneum's expansive nature and its sneaky growth, these tumours frequently show up big and with vague symptoms, including discomfort, distension in the abdomen, or coincidental imaging abnormalities [3].

The most important determinant affecting long-term survival is total excision, and surgical resection is still the major treatment option, particularly for localised disease. Because of their size, intricate anatomical linkages, and risk for malignancy, open surgery has historically been the accepted treatment for these tumors [4]. However, advances in minimally invasive surgery have made laparoscopic resection a feasible option in selected patients [5]. The indications for the laparoscopic approach to the retroperitoneal area have recently expanded to include pancreatic and aortic procedures, thanks to advancements in laparoscopic methods and equipment [6-9].

Previous systematic reviews and meta-analyses, such as those by Constantinides et al. (2012) [10] and Fan et al. (2012) [11], have compared laparoscopic with retroperitoneoscopic approaches for adrenalectomy and nephrectomy, respectively, consistently reporting benefits such as shorter hospital stays, faster recovery, and comparable safety and oncological outcomes. However, these reviews are limited to adrenal and renal surgeries and do not address the broader spectrum of RPTs, particularly those of varied histologies, including primary RPTs and metastatic lesions requiring complex resections like lymphadenectomies. To our knowledge, no systematic review has comprehensively evaluated the safety, feasibility, and clinical outcomes of laparoscopic RPT resections beyond the adrenal and renal context. Therefore, this systematic review aims to synthesize the current evidence on the use of laparoscopic approaches in the management of RPTs, both benign and malignant, highlighting operative outcomes, complications, recurrence, and functional recovery to inform surgical decision-making in more complex retroperitoneal pathology.

## Review

Methods

Study Design

This systematic review was conducted in accordance with the Preferred Reporting Items for Systematic Reviews and Meta-Analyses (PRISMA) 2020 guidelines [12]. The objective of the review was to assess the feasibility, safety, and surgical outcomes of laparoscopic resection in managing large-volume, non-adrenal and non-renal RPTs.

Eligibility Criteria

Inclusion criteria: The selection of studies for this review was based on predefined eligibility criteria. Adult patients (≥18 years) with a diagnosis of primary RPTs, excluding those of adrenal or renal origin, were considered. Studies were required to evaluate laparoscopic surgical resection, with or without hand-assisted or robotic techniques. Comparators included open surgery, conventional approaches, or no comparator in single-arm designs. Eligible studies were those reporting at least one clinical outcome, such as operative time, intraoperative blood loss, hospital stay, complication rate, conversion rate, recurrence, or long-term survival. Only randomized controlled trials, prospective or retrospective cohort studies, and case series with a minimum of five patients were included. Furthermore, only studies published in the English language were considered.

Exclusion Criteria: Case reports, case series with fewer than five patients, studies focusing exclusively on adrenal or renal tumors, pediatric populations, and non-English studies were excluded.

Information Sources and Search Strategy

A comprehensive literature search was conducted across four major electronic databases: PubMed, ScienceDirect, Google Scholar, and the Cochrane Library, encompassing studies published up to August 1, 2025. The search combined controlled vocabulary (MeSH (Medical Subject Headings) terms) and free-text keywords related to the surgical approach, tumor location, and specific tumor histologies. For PubMed, the search string included:

("Laparoscopic Surgery"[Mesh] OR "laparoscopic resection" OR "laparoscopic excision" OR "laparoscopy") AND ("Retroperitoneal Neoplasms"[Mesh] OR retroperitoneal tumor* OR retroperitoneal mass* OR retroperitoneal neoplasm*) AND (ganglioneuroma OR schwannoma OR neurofibroma OR paraganglioma OR liposarcoma OR leiomyosarcoma OR fibrosarcoma OR fibroma OR hemangioma OR teratoma) NOT (adrenal OR renal).

Similar search strategies were adapted for ScienceDirect and Google Scholar using appropriate Boolean operators and syntax.

Study Selection

The titles and abstracts of all retrieved records were screened for relevance. Full-text articles were subsequently assessed against predefined inclusion and exclusion criteria. The inclusion and exclusion criteria were applied.

Data Extraction

Data extraction was carried out using a pre-designed standardized form. Extracted data included the author, year, study design, country, patient population, tumor characteristics (type and size), surgical approach (laparoscopic, open, hand-assisted, robotic), and perioperative outcomes such as operative time, blood loss, complication rate, hospital stay, recurrence, and need for conversion. For studies directly comparing laparoscopic and open or conventional surgical techniques, statistical results of relevant outcomes were also extracted.

Risk of Bias Assessment

The methodological quality of the included non-randomized studies was assessed using the ROBINS-I (Risk of Bias In Non-randomized Studies of Interventions) tool [13]. This assessment evaluated potential bias across several domains, including confounding, selection, classification of interventions, deviations from intended interventions, missing data, measurement of outcomes, and selection of reported results. Each study was rated as having low, moderate, serious, or critical risk of bias accordingly.

Data Synthesis

A narrative synthesis was undertaken to summarize the findings from all included studies. Descriptive data from non-comparative case series and cohort studies were synthesized to provide an overview of the feasibility, safety, and clinical outcomes of laparoscopic resection for RPTs. For the three studies that included a comparator group (laparoscopic vs. open or conventional surgery), statistical data on perioperative outcomes such as operative time, blood loss, complications, hospital stay, and recurrence were also tabulated to facilitate comparison. Statistical data on perioperative outcomes for non-comparative studies were also tabulated. Due to the limited number of comparative studies and the heterogeneity of tumor types, surgical techniques, and outcome measures, a formal meta-analysis was not performed.

Results

**Figure 1:**
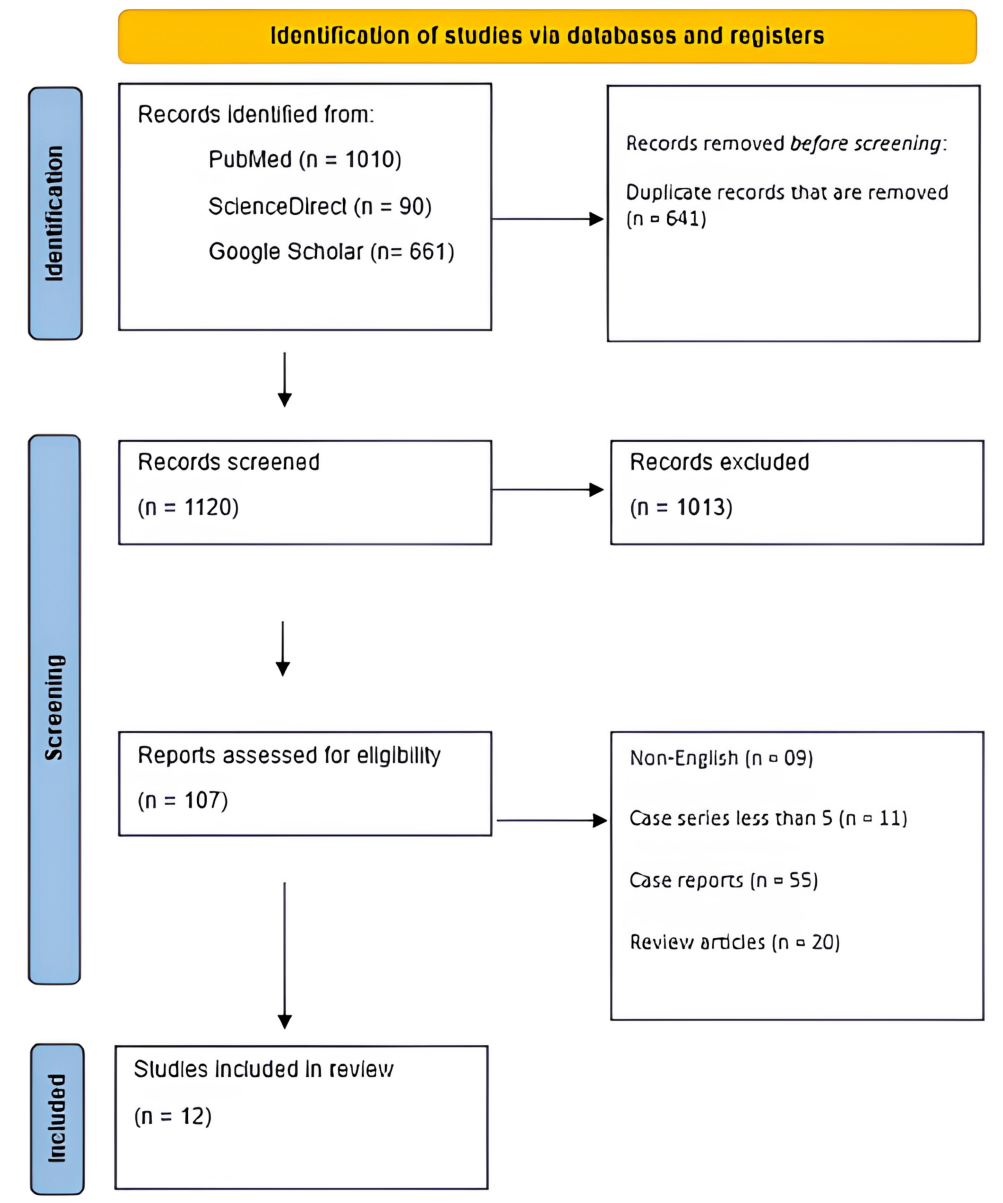
PRISMA flow diagram PRISMA: Preferred Reporting Items for Systematic Reviews and Meta-Analyses

A total of 1,761 records were identified through database searches, including PubMed (n = 1,010), ScienceDirect (n = 90), and Google Scholar (n = 661). After removal of 643 duplicate records, 1,120 studies remained for screening. Following title and abstract screening, 1,013 records were excluded for irrelevance, leaving 107 articles for full-text assessment. Of these, 95 studies were excluded for the following reasons: non-English language (n = 9), case series with fewer than five patients (n = 11), single case reports (n = 55), and review articles (n = 20). Ultimately, 12 studies met the inclusion criteria and were included in the qualitative synthesis (Figure 1). Characteristics and main findings are described in Table 1. Statistical outcomes of non-comparative studies are stated in Table 2 and of comparative studies in Table 3.

**Table 1:** Characteristics and main findings of the included studies. NSGCT: non-seminomatous germ cell tumor; RPLND: retroperitoneal lymph node dissection; EBL: estimated blood loss; RRTM: residual retroperitoneal tumor mass; RRRTM: resection of a residual retroperitoneal tumor mass

Author(s)	Year	Study Design	Population (n)	Age (Range/Mean)	Tumor Type	Tumor Size (cm)	Surgical Approach	Complications	Outcomes Assessed	Results
Shalhav et al. [14]	1999	Retrospective comparative case series	4 laparoscopy vs 4 open	50–62 yrs	Metastatic adenocarcinoma of ovary, sclerosing lymphoma, follicular lymphoma, transitional cell carcinoma (TCC), myxoid leiomyosarcoma, leiomyosarcoma, metastatic seminoma, mature metastatic teratoma.	Laparoscopic: 3.7 (2–6); Open: 6.5 (1–10)	Laparoscopic vs open exploration	1 (lap), 3 (open)	Diagnostic yield, blood loss, complications, hospital stay, pain medication, diet, ambulation	Compared to open surgery, the laparoscopic group had significantly lower blood loss (90 vs 440 mL), smaller hematocrit drop (5.1% vs 7.8%), earlier diet resumption (3 vs 5 days), lower morphine use (128 vs 161 mg), faster ambulation (2.3 vs 6 days), and shorter hospital stay (4.8 vs 6 days), though with longer operative time (7.8 vs 4.3 hours). No operative mortality occurred.
Tsivian et al. [15]	2009	Retrospective case series	8	46–73 yrs	RCC recurrence (3), Lymphoma (1), Sarcoma (1), Schwannoma (1), Cyst (2)	Average lesion size (largest diameter on imaging) was 6.88 cm ranging from 2 to 16 cm.	Laparoscopic excision/biopsy	1 bowel injury (repaired); No conversions	Operative time, blood loss, complications, hospital stay, pathology	All cases were completed laparoscopically with no conversions. The mean operative time was 131 minutes, and blood loss ranged from 0 to 200 mL, with no transfusions required. One bowel injury was repaired intraoperatively. Recovery was uneventful; hospital stay was 2–7 days. Pathology revealed benign and malignant lesions. No recurrences occurred over >2 years of follow-up.
Ahn et al. [16]	2011	Retrospective cohort study	20	Median 45.5 yrs	Lymphangioma (7), Ganglioneuroma (3), Schwannoma (2), Paraganglioma (2), Castleman disease (2)	2.0–9.5 (median 4.7)	Laparoscopic resection	2 complications (10%, both conservative)	Feasibility, safety, operative time, complications, pathology, vessel adherence	All tumors were resected laparoscopically except one conversion due to bleeding. Median operative time was 117.5 minutes; blood loss was low (median 50 mL). Two postoperative complications occurred, managed conservatively. Tumor size and vessel adherence had no significant effect on outcomes. Median hospital stay was 5 days.
Kira et al. [17]	2024	Retrospective case series	9 patients with benign retroperitoneal tumors	Median 44 (15–70)	Paraganglioma, Ganglioneuroma, Schwannoma	Median 3.0 (1.8–12)	Retroperitoneal laparoscopic resection	None ≥ Clavien–Dindo grade 3	Operative time, blood loss, complications, margin status	Median operative time was 144 minutes; blood loss was low (median 7 mL). All resections had negative margins; no major complications or conversions reported.
Ji et al. [18]	2015	Retrospective comparative cohort	26 patients: 13 laparoscopic vs 13 open surgery	Mean: 48.46 years	Benign neurilemmoma	Mean: 5.39	Laparoscopic vs open resection	No major complications reported	Operative time, EBL, transfusion, recurrence, hospital stay, complications	No significant difference in EBL, transfusion, or recurrence. Hospital stay was significantly shorter in the laparoscopic group (4.5 vs 7 days; p=0.031). Complete tumor resection achieved.
Rassweiler et al. [19]	1996	Prospective case series	26 NSGCT patients (17 Stage I, 9 post-chemo Stage IIb/IIc)	Not reported	NSGCT	Not specified	Laparoscopic RPLND (modified template)	1 ureteral stricture, 1 pulmonary embolism, 1 retrograde ejaculation	Feasibility, complications, recurrence	Laparoscopic resection was successfully completed in 16/17 stage I cases. Embryonal carcinoma was found in 1 patient. For stage II disease, laparoscopy was successful in only 2/9 cases; most required conversion to open surgery. No regional relapses were noted, though 2 patients had pulmonary metastases successfully treated with chemotherapy.
Öztürk et al. [20]	2012	Prospective cohort	29 post-chemotherapy NSTGCT patients with RRTM <5 cm	Median 25 (16–59)	NSGCT	Median 2.1 (1.1–4.7)	Laparoscopic RRRTM	2 conversions due to bleeding, 1 due to obesity, 1 hand-assisted procedure	Feasibility, conversion, histopathology, recurrence	Laparoscopic resection was successful in 25/29 patients (86%), with minimal complications. Histology showed 55% mature teratoma, 41% necrosis, and 3% viable tumor. One early relapse occurred within 1 month, while the overall follow-up period showed promising oncologic control.
Öztürk et al [21]	2019	Retrospective comparative cohort	150 post-chemotherapy NSTGCT patients (89 laparoscopic, 61 open)	median age 27 [range 16–66] years	NSGCT	Median 2.0 (Laparoscopic), 4.2 (Open)	L-RRRTM vs. C-RRRTM	Laparoscopic group: 6% perioperative, 19% postoperative; Open group: 12% perioperative, 30% postoperative	Recurrence, complications, hospital stay, and operative duration	Laparoscopic approach showed significantly better outcomes in terms of recurrence (9% vs. 31%), shorter operative time (156 vs. 221 min), and reduced hospital stay (2 vs. 6 days). Conversion rate was 15% in the laparoscopic group. Both groups had acceptable complication profiles.
Li et al. [22]	2011	Retrospective case series	131 patients with pheochromocytoma (120 adrenal, 11 extra-adrenal)	8–77 years	Adrenal and extra-adrenal pheochromocytoma	Mean: 4.2 (Stage 1), 5.6 (Stage 2), 6.7 (Stage 3)	Retroperitoneal laparoscopic resection	3 recurrences; no distant metastases or deaths	Operative time, intraoperative blood loss, recurrence, follow-up (1–70 months)	Operative time reduced from 105 to 75 minutes and blood loss from 450 mL to 70 mL across experience stages. Tumor size increased with experience but without affecting safety. Recurrence occurred in 3/131 cases (2.3%); no metastases or mortality observed.
Steiner et al. [23]	2004	Retrospective cohort (10-year single-center)	185 NSGCT patients (188 L-RPLND procedures)	Not specified	Nonseminomatous germ cell tumor	Not specified (Stage I & II low-volume)	Laparoscopic RPLND	Conversion rate 2.6%; minimal morbidity; ejaculation preserved in 98.4%	Operative time, blood loss, active tumor presence, relapse, ejaculation preservation, survival	Mean operative time: 256 min (Stage I), 243 min (Stage II); mean blood loss: 159 mL (Stage I), 78 mL (Stage II). Active tumor in 19.5% (Stage I) and 50% (clinical Stage IIA). 6 relapses; 0 deaths. Ejaculation preserved in 98.4%.
Albqami and Janetschek [24]	2005	Retrospective case series (13-year experience)	162 testicular cancer patients (103 Stage I, 59 Stage II [43 IIB, 16 IIC])	Mean age was 29.2 years (range 15–56 years	Testicular cancer (NSGCT)	6 cm	Laparoscopic RPLND	3 conversions (Stage I); low complication rate	Operative time, blood loss, relapse, feasibility post-chemo, follow-up (6–113 months)	Mean operative time: 217 minutes (Stage I), 216 min (IIB), 281 min (IIC); blood loss: 144 mL (Stage I), 165 mL (Stage II). Relapse: 2 retroperitoneal (1.2%), 4 distant (2.5%). Total relapse rate: 3.7%.
Zhang and Xiu [25]	2011 (study period: 2007–2009)	Retrospective case series	14 patients with primary retroperitoneal tumors	Median 44 years	Primary retroperitoneal tumors (includes epithelioid hemangioma)	Median 7.6 cm	Laparoscopic (n=11), hand-assisted (n=2), 1 conversion to open	No major complications; no transfusions	Feasibility, operative time, blood loss, complications, recurrence	Median operative time 139 minutes; mean EBL 59.2 mL; 1 conversion; no recurrence at 17-month follow-up

**Table 2:** Statistical outcome measures of non-comparative studies.

Author(s)	Operative Time (minutes)	Blood Loss (mL)	Hospital Stay (days)	Conversion Rate (%)	Complication Rate (%)	Recurrence Rate (%)
Tsivian et al. [15]	131 (mean)	0–200 (range)	2–7 (range)	0	1/8 (12.5%)	0
Ahn et al. [16]	117.5 (median)	50 (median)	5 (median)	1/20 (5%)	2/20 (10%)	0
Kira et al. [17]	144 (median)	7 (median)	Not reported	0	0 ≥ Clavien-Dindo grade 3	0
Rassweiler et al. [19]	243–256	78–159	Not reported	Stage I: low; Stage II: high	3/26 (11.5%)	0 regional; 2 pulmonary
Öztürk et al. (2012) [20]	Not reported	Not reported	Not reported	4/29 (14%)	Low	1 relapse (<1 month)
Li et al. [22]	75–105 (depending on experience)	70–450	Not reported	0	3 recurrences in 131 (2.3%)	2.3%
Steiner et al. [23]	243–256	78–159	Not reported	2.6%	Minimal; ejaculation preserved (98.4%)	6 relapses (3.2%)
Albqami and Janetschek [24]	217–281	144–165	Not reported	3 (Stage I)	Low	3.7%
Zhang and Xiu [25]	139 (median)	59.2 (mean)	Not reported	1/14 (7.1%)	None	0

**Table 3:** Statistical outcome measures of comparative studies.

Author	Operative Time (minutes)	Blood Loss (mL)	Hospital Stay (days)	Complication Rate (%)	Recurrence Rate (%)	Conversion Rate (%)
Shalhav et al. (1999) [14]	468 (7.8 h) lap vs 258 (4.3 h) open	90 (lap) vs 440 (open)	4.8 (lap) vs 6.8 (open)	0% transfusion in both, lower morphine needs, faster ambulation & diet	Not reported	0
Ji et al. (2015) [18]	169.5 ± 78.1 (lap) vs 158.7 ± 62.3 (open)	101.3 ± 125.5 (lap) vs 487 ± 855.8 (open), NS	4.5 ± 2.16 (lap) vs 7.0 ± 3.43 (open)*	12.5% (lap) vs 0% (open), NS	0% in both groups	0
Öztürk et al. (2019) [21]	156 (45–341) lap vs 221 (95–792) open	Not reported	2 (1–13) lap vs 6 (3–26) open	19% (lap) vs 30% (open) (post-op complications)	9% (lap) vs 31% (open)	15% (lap group)

**Figure 2:**
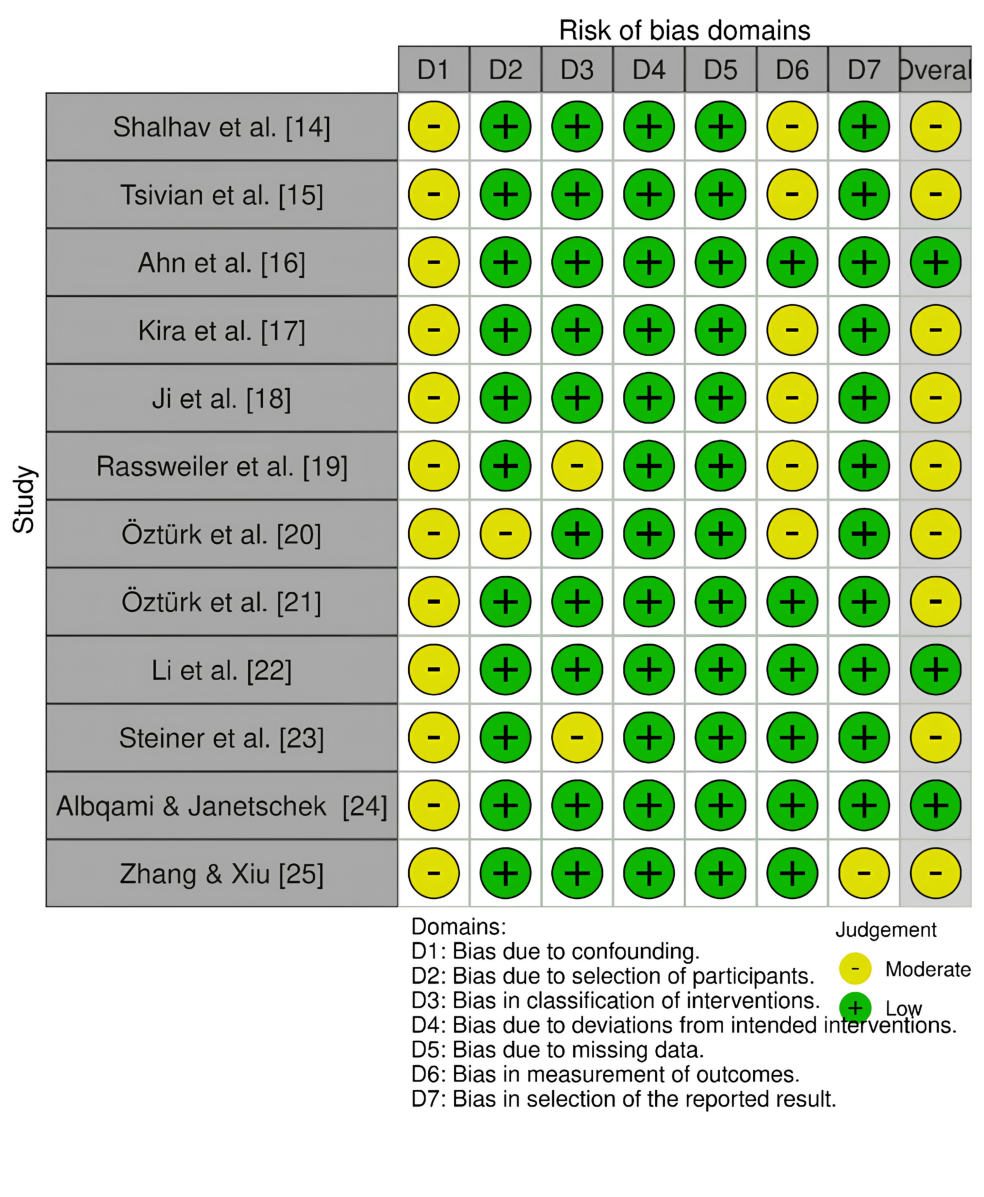
Quality assessment of studies by ROBINS-I tool. References: [14=25] ROBINS-I: Risk Of Bias In Non-randomised Studies - of Interventions

The quality assessment of the included non-randomized studies was conducted using the ROBINS-I tool, which evaluates bias across seven domains (Figure 2). The overall assessment revealed that most studies demonstrated low risk of bias across the majority of domains. However, moderate risk was commonly identified in Domain 1 (confounding) and Domain 6 (measurement of outcomes), as these are frequently impacted in observational designs due to a lack of control over baseline characteristics and reliance on retrospective data collection.

Operative Time

Operative time varied across studies and tumor types. Shalhav et al. (1999) found longer operative time in the laparoscopic group compared to open surgery (7.8 vs 4.3 hours) [14]. Tsivian et al. (2009) reported a mean time of 131 minutes [15], and Ahn et al. (2011) reported a median of 117.5 minutes [16]. Kira et al. (2024) noted a median of 144 minutes [17]. Ji et al. (2015) did not report specific values for operative time differences but included them in feasibility outcomes [18]. Rassweiler et al. (1996) found mean times of 256 minutes (Stage I) and 243 minutes (Stage II) for laparoscopic-retroperitoneal lymph node dissection (L-RPLND) [19]. Albqami and Janetschek (2005) reported mean times of 217 minutes (Stage I), 216 minutes (Stage IIB), and 281 minutes (Stage IIC) [24]. Li et al. (2011) noted that operative time decreased from 105 to 75 minutes with experience [22]. Öztürk et al. (2019) found that laparoscopic RRRTM had shorter operative duration than open (156 vs 221 minutes), while Öztürk et al. (2012) did not report operative time numerically [20-21]. Zhang and Xiu (2011) reported a median operative time of 139 minutes for laparoscopic PRT resection [25].

Blood Loss and Transfusion

Laparoscopic surgery was consistently associated with reduced blood loss. Shalhav et al. (1999) found significantly less blood loss in the laparoscopic group (90 vs 440 mL) [14]. Tsivian et al. (2009) noted blood loss of 0-200 mL without transfusions [15]. Ahn et al. (2011) reported a median blood loss of 50 mL [16], while Kira et al. (2024) showed extremely low median loss (7 mL) [17]. Ji et al. (2015) reported no significant difference between groups [18]. Rassweiler et al. (1996) reported 159 mL (Stage I) and 78 mL (Stage II) [19], while Albqami and Janetschek (2005) reported 144 mL (Stage I) and 165 mL (Stage II) [24]. Li et al. (2011) demonstrated a reduction from 450 to 70 mL with experience [22]. Öztürk et al. (2019) did not provide absolute values but favored laparoscopy in perioperative metrics [20,21]. Zhang and Xiu (2011) reported low estimated blood loss with an average of 59.2 mL and no need for blood transfusions [25].

Complications and Conversions

Complication rates were generally low, showing the safety of the procedure. Shalhav et al. (1999) reported one vs three complications (laparoscopic vs open) [14]. Tsivian et al. (2009) had one bowel injury, no conversions [15]. Ahn et al. (2011) reported two conservatively managed complications and one conversion due to bleeding [16]. Kira et al. (2024) had no ≥ Grade 3 complications [17]. Ji et al. (2015) reported no major complications [18]. Rassweiler et al. (1996) reported ureteral stricture, pulmonary embolism, and retrograde ejaculation [19]. Öztürk et al. had four conversions (three due to technical difficulty, one hand-assisted) in their 2012 study [20], and noted perioperative and postoperative complication rates of 6% and 19% (laparoscopic) vs 12% and 30% (open) in their 2019 study [21]. Albqami and Janetschek (2005) had three conversions (Stage I) and low complication rates [24]. Li et al. (2011) reported three recurrences but no deaths or major complications [22]. Zhang and Xiu (2011) reported no major complications and one conversion to laparotomy due to a case of epithelioid hemangioma [25].

Hospital Stay and Recovery

The laparoscopic approach led to quicker recovery. Shalhav et al. (1999) found shorter hospital stay (4.8 vs 6 days), faster ambulation (2.3 vs 6 days), and earlier diet resumption (3 vs 5 days) with less morphine use (128 vs 161 mg) [14]. Tsivian et al. (2009) reported a 2-7 day stay [15], and Ahn et al. (2011) reported a median of 5 days [16]. Ji et al. (2015) found significantly shorter hospital stay in the laparoscopic group (4.5 vs 7 days, p=0.031) [18]. Öztürk et al. (2019) also showed a shorter stay with laparoscopy (2 vs 6 days) [21].

Feasibility and Conversion Rates

Feasibility was consistently high in early-stage and benign cases. Tsivian et al. (2009) [15], Ahn et al. (2011) [16], and Kira et al. (2024) [17] completed nearly all procedures laparoscopically. Rassweiler et al. (1996) succeeded in 16/17 Stage I non-seminomatous germ cell tumor (NSGCT) cases, but only 2/9 post-chemotherapy Stage II patients, suggesting limitations [19]. Öztürk et al. had a success rate of 86% in 2012 [20], and had a 15% conversion rate in 2019 [21]. Albqami and Janetschek (2005) demonstrated success across a broader range, including post-chemo patients [24]. Zhang and Xiu (2011) successfully completed laparoscopic resection in 13 of 14 patients (93%) using either a pure laparoscopic or hand-assisted approach [25].

Oncologic Outcomes and Recurrence

Oncologic control was adequate in well-selected cases. Shalhav et al. (1999) achieved diagnostic yield in all cases [14]. Tsivian et al. (2009) [15] and Ahn et al. (2011) [16] found no recurrences. Ji et al. (2015) [18] and Kira et al. (2024) [17] achieved complete resections. Rassweiler et al. (1996) observed no regional relapses but two pulmonary metastases [19]. Öztürk et al. (2012) had one early relapse [20]. Öztürk et al. (2019) showed lower recurrence with laparoscopy (9% vs 31%) [21]. Li et al. (2011) observed a 2.3% recurrence rate with no metastases [22]. Albqami and Janetschek (2005) reported a 3.7% total relapse rate [24]. Zhang and Xiu (2011) observed no recurrences during a median follow-up of 17 months, supporting the oncologic adequacy of laparoscopic PRT resection [25].

Discussion

This systematic review demonstrates that laparoscopic resection is a safe and feasible approach for the management of large-volume, non-adrenal and non-renal RPTs across a range of histologies, with acceptable complication rates, good oncologic outcomes, and benefits in perioperative recovery. Across the included studies, laparoscopic approaches were consistently feasible and associated with favorable perioperative morbidity compared with open surgery in carefully selected patients. Several comparative series found reduced blood loss, shorter hospital stay, earlier return to diet and ambulation, and lower analgesic requirements for laparoscopic cases despite occasionally longer operative times [14,18,21]. For example, Shalhav et al. (1999) reported markedly lower mean blood loss (90 vs 440 mL), shorter length of stay, and reduced opioid use in the laparoscopic group [14], while Ji et al. (2015) found a significantly shorter hospital stay after laparoscopic resection [18]. These consistent perioperative advantages were echoed in single-arm series reporting low median blood loss and short length of stay [15-17,25].

When oncologic outcomes were reported, laparoscopic resection achieved acceptable short- to mid-term control in appropriately selected patients. Several series of NSGCT and post-chemotherapy residual masses reported low regional relapse rates and preserved oncologic control comparable with open approaches in early or low-volume disease [19,21,23,24]. For retroperitoneal benign and primary tumors, negative margin rates and low recurrence were reported [17,22,25]. However, interpretation must remain cautious: many studies excluded large, invasive, or post-chemotherapy fibrotic tumors that are technically challenging and more likely to require conversion or open resection [19,20]. Indeed, Rassweiler et al. [19] and Öztürk et al. [20] highlight reduced feasibility for bulky or heavily fibrotic post-chemotherapy masses, underscoring that oncologic safety appears contingent on careful patient selection and tumor characteristics.

Reported major complication rates were generally low. The series described occasional intraoperative injuries (e.g., bowel injury [15], ureteral stricture, and pulmonary embolism [19]) and modest conversion rates that correlated with tumor size, prior chemotherapy, bleeding, and patient body habitus [20,21]. Functional preservation (e.g., ejaculatory function after RPLND) was high in dedicated series [23]. Overall, laparoscopy demonstrated a favorable morbidity profile when performed in centers with appropriate expertise.

Several studies in this review demonstrate that L-RPLND is a safe and effective option in selected patients with NSGCT, particularly in early-stage or post-chemotherapy settings, with favorable oncologic and functional outcomes [20,21,23,24]. In addition, laparoscopic resection has shown promising results in both benign and malignant PRTs, with high resection success rates, minimal complications, and low recurrence in tumors up to 12 cm [16,17,25]. Furthermore, its applicability across heterogeneous retroperitoneal pathologies is supported by studies reporting reduced blood loss, shorter hospital stays, and faster recovery compared to open surgery [14,15].

The aggregate evidence supports laparoscopic resection as a safe and effective option for selected patients with non-adrenal, non-renal retroperitoneal tumors, particularly small to moderate-sized, well-circumscribed lesions and early-stage NSGCT or benign neoplasms, when performed by surgeons with appropriate oncologic and advanced laparoscopic expertise [14-18,21]. Contraindications or caution are indicated for bulky, invasive, or heavily post-treatment fibrotic masses where conversion rates and complications rise [19,20]. Shared decision-making should consider tumor biology, prior treatments, anatomic relationships to major vessels, and institutional experience.

This review is limited primarily by the nature and quality of the available evidence. Most included studies were retrospective case series or single-center cohort studies, with only a small number of comparative analyses, which increases the potential for selection bias and limits the ability to draw definitive causal inferences. The ROBINS-I assessment indicated that several studies were at moderate to serious risk of bias due to confounding, patient selection, and incomplete outcome reporting. There was marked heterogeneity in tumor types (benign vs malignant, primary vs post-chemotherapy residual), tumor size definitions, and surgical approaches (pure laparoscopic, retroperitoneal, transperitoneal, hand-assisted), as well as in surgeon experience. Reporting of long-term oncologic outcomes, recurrence rates, and margin status was incomplete in several series, and follow-up duration varied widely, limiting the ability to assess durable oncologic safety. Furthermore, perioperative outcomes such as blood loss, complications, and functional results were not uniformly defined or graded (e.g., use of Clavien-Dindo classification was inconsistent). Language restriction to English and the exclusion of case series with fewer than five patients may have led to publication bias.

## Conclusions

Based on the available evidence, laparoscopic resection appears to be a safe and effective approach for managing large-volume non-adrenal and non-renal RPTs, offering consistent perioperative benefits such as reduced blood loss, shorter hospital stay, and faster recovery. Across all included studies, outcomes were favorable for the laparoscopic technique, and in the subset of studies directly comparing it to open surgery, the laparoscopic approach demonstrated superior perioperative results without compromising oncological safety. These findings support the feasibility and potential advantages of laparoscopic surgery in appropriately selected patients, although further high-quality comparative studies are warranted to strengthen the evidence base.
